# Retraction Note: Effect of sub-dermal exposure of silver nanoparticles on hepatic, renal and cardiac functions accompanying oxidative damage in male Wistar rats

**DOI:** 10.1038/s41598-026-52159-6

**Published:** 2026-05-12

**Authors:** Janet Olayemi Olugbodi, Bashir Lawal, Godiya Bako, Amos Sunday Onikanni, Sulama M. Abolenin, Soliman S. Mohammud, Farid S. Ataya, Gaber El‑Saber Batiha

**Affiliations:** 1 Department of Biochemistry, Bingham University, Abuja‑Keffi Expressway Road, P.M.B 005, Karu, Nigeria; 2 Department of Pathology, University of Pittsburgh, Pittsburgh, United States; 3 UPMC Hillman Cancer Center, University of Pittsburgh, Pittsburgh, United States; 4 Biochemistry Unit, Department of Chemical Sciences, Afe Babalola University, Ado‑Ekiti, Ekiti State Nigeria; 5 College of Medicine, Graduate Institute of Biomedical Science, China Medical University, Taichung, Taiwan; 6 Biology Department, Thurobah University College, Thurobah, Republic of Congo; 7 Department of Biochemistry, College of Science, King Saud University, P. O. Box 2455, 11451 Riyadh, Saudi Arabia; 8 Department of Pharmacology and Therapeutics, Faculty of Veterinary Medicine, Damanhour University, Damanhour, 22511 AlBeheira Egypt

Retraction of: *Scientific Reports* 10.1038/s41598-023-37178-x, published online 29 June 2023

The Editor has retracted this Article.

After publication, concerns were raised regarding the figures. Figure 8, Heart, panels E and F were found to overlap, for which a correction was issued. Subsequently, Figure 8 panels A and D were also found to overlap with rotation. The Editors requested all original data underlying the paper, but the data provided by the Authors could not be validated. The Editors have lost confidence in the data and conclusions of this Article.

The Authors did not respond to correspondence from the Editors regarding this retraction.

